# Neural Substrates of Social Emotion Regulation: A fMRI Study on Imitation and Expressive Suppression to Dynamic Facial Signals

**DOI:** 10.3389/fpsyg.2013.00095

**Published:** 2013-02-27

**Authors:** Pascal Vrticka, Samanta Simioni, Eleonora Fornari, Myriam Schluep, Patrik Vuilleumier, David Sander

**Affiliations:** ^1^Center for Interdisciplinary Brain Sciences Research, Department of Psychiatry and Behavioral Sciences, School of Medicine, Stanford UniversityStanford, CA, USA; ^2^Swiss Center for Affective Sciences, University of GenevaGeneva, Switzerland; ^3^Laboratory for the study of Emotion Elicitation and Expression (E3 Lab), Department of Psychology, University of GenevaGeneva, Switzerland; ^4^Service of Neurology, Department of Clinical Neurosciences, Centre Hospitalier Universitaire Vaudois and Lausanne University HospitalLausanne, Switzerland; ^5^Centre d’Imagerie BioMédicale, Radiology Department, Centre Hospitalier Universitaire Vaudois and University of LausanneLausanne, Switzerland; ^6^Laboratory for Behavioral Neurology and Imaging of Cognition, Department of Neuroscience, University Medical Center, University of GenevaGeneva, Switzerland

**Keywords:** emotion regulation, expressive suppression, imitation, emotional facial expressions, fMRI

## Abstract

Emotion regulation is crucial for successfully engaging in social interactions. Yet, little is known about the neural mechanisms controlling behavioral responses to emotional expressions perceived in the face of other people, which constitute a key element of interpersonal communication. Here, we investigated brain systems involved in social emotion perception and regulation, using functional magnetic resonance imaging (fMRI) in 20 healthy participants. The latter saw dynamic facial expressions of either happiness or sadness, and were asked to either imitate the expression or to suppress any expression on their own face (in addition to a gender judgment control task). fMRI results revealed higher activity in regions associated with emotion (e.g., the insula), motor function (e.g., motor cortex), and theory of mind (e.g., [pre]cuneus) during imitation. Activity in dorsal cingulate cortex was also increased during imitation, possibly reflecting greater action monitoring or conflict with own feeling states. In addition, premotor regions were more strongly activated during both imitation and suppression, suggesting a recruitment of motor control for both the production and inhibition of emotion expressions. Expressive suppression (eSUP) produced increases in dorsolateral and lateral prefrontal cortex typically related to cognitive control. These results suggest that voluntary imitation and eSUP modulate brain responses to emotional signals perceived from faces, by up- and down-regulating activity in distributed subcortical and cortical networks that are particularly involved in emotion, action monitoring, and cognitive control.

## Introduction

During the last decade, studies using functional magnetic resonance imaging (fMRI) have begun to disclose the neural substrates of distinct emotion regulation strategies in response to various affective stimuli. In this context, the *process model of emotion* proposed by Gross (1998) has provided a major psychological theoretical framework that distinguishes between antecedent-versus response-focused emotion regulation, often operationalized as (cognitive) re-appraisal versus (expressive) suppression. Antecedent-focused emotion regulation was further extended by additional components such as *situation selection and modification* as well as *attention deployment*. These are thought to affect emotion processing even earlier than re-appraisal through avoidance or modification of, or distraction from an emotion-eliciting situation (Gross, 2002).

Several imaging studies have tested for brain activation differences between natural viewing (no explicit emotion regulation) versus re-appraisal (Ochsner et al., 2002, 2004; Ochsner and Gross, 2005; Kim and Hamann, 2007), or between natural viewing versus suppression (Levesque et al., 2003); while other investigations compared different emotion regulation strategies with each other, particularly re-appraisal versus suppression (Goldin et al., 2008; Vrticka et al., 2011). Furthermore, a few recent studies focused on the difference between attention deployment (also referred as to distraction) versus re-appraisal (McRae et al., 2010; Kanske et al., 2011; Payer et al., 2012). Most of these studies on emotion regulation examined modulation of brain responses to complex visual scenes or movie excerpts. Taken together, results converge to indicate that emotion regulation skills rely on a number of prefrontal cortical areas, either implicated in top-down modulation of limbic regions, or more generally involved in attention selection, action or thought inhibition, and working memory. In addition, both re-appraisal and distraction have been found effective in down-regulating neural responses in brain areas critically involved in the processing of emotional stimuli (such as amygdala or insula), which are activated otherwise during natural viewing conditions. These findings have been used to suggest that antecedent-focused emotion regulation strategies provide a beneficial means of controlling one’s emotions, particularly in the case of re-appraisal (Gross, 1998, 2002; McRae et al., 2010). Less consistent results have been reported for response-focused emotion regulation and in particular (expressive) suppression, which is generally regarded as a less efficient strategy for emotion control (Gross, 1998, 2002). It may actually be associated with increased activity in emotion brain regions, such as the insula or amygdala (Goldin et al., 2008), or produce decreases in only some of these areas under specific circumstances (Vrticka et al., 2011).

Social reactions to others may not only involve the ability to express or – in some circumstances – suppress our own emotions, but also imply the sharing of others’ feelings. Research on empathy suggests that facial mimicry, possibly associated with “mirror” neural activity in the observer, may constitute an important feature of social processing and social emotional understanding (Premack and Woodruff, 1978; Rizzolatti et al., 1996; Leslie et al., 2004; Lee et al., 2006; Pfeifer et al., 2008). The extensive brain network(s) of such sharing have been particularly investigated in experimental paradigms involving empathy for pain (see Singer and Lamm, 2009; Lamm et al., 2011). This has lead to the description of a “core network” of affective (pain) empathy, comprising anterior insula (aINS), and anterior cingulate cortex. It has been suggested that picture-based paradigms of pain observation may also reveal stronger somatosensory area activity in the observer (Singer and Lamm, 2009; Corradi-Dell’Acqua et al., 2011; Lamm et al., 2011). Furthermore, when more abstract visual stimuli were used to provide information about other’s feelings, increased activity has been observed in brain areas that are typically associated with theory of mind, such as the precuneus, ventral medial prefrontal cortex, superior temporal cortex, and temporo-parietal junction (Lamm et al., 2011). Recently, activity within this extended affective empathy network and “mirror neuron system” has also been described during automatic and spontaneous facial mimicry of happy, sad, and angry expressions (Likowski et al., 2012).

Building on such evidence from research on emotion regulation, empathy, and facial mimicry, we designed an fMRI study to specifically examine the social aspects of emotion perception and regulation. This included the introduction of two novel experimental factors.

Firstly, emotions to be regulated were not induced by images or movie-clips of complex scenes [e.g., pictures from the International Affective Picture System (IAPS) or movies of food or disgusting places], as used, to the best of our knowledge, in all fMRI studies on emotion regulation so far (e.g., Ochsner et al., 2002, 2004; Ochsner and Gross, 2005; Kim and Hamann, 2007; Goldin et al., 2008; McRae et al., 2010; Kanske et al., 2011; Vrticka et al., 2011; Payer et al., 2012), but rather by short movie-clips of actors displaying happy or sad facial expressions. Faces represent a category of stimuli with major social significance, and regulating one’s emotion in response to others’ facial expressions is a crucial ability during social interactions. In a previous fMRI study, we demonstrated distinctive patterns of regulation between social versus non-social emotion conditions (Vrticka et al., 2011), but the social nature of stimuli in the latter study was essentially defined by the presence of humans in complex visual scenes – not faces specifically. Here, by using face movies, we could test for emotion regulation at a level closer to direct real-world interpersonal interaction.

Secondly, the experimental conditions used in our study differed from more recent fMRI studies on emotion regulation (Ochsner et al., 2002, 2004; Ochsner and Gross, 2005; Kim and Hamann, 2007; Goldin et al., 2008; McRae et al., 2010; Kanske et al., 2011; Vrticka et al., 2011; Blechert et al., 2012; Payer et al., 2012), again inspired by the above-mentioned fact that social emotional understanding (at least partly) involves facial mimicry possibly linked with mirror neuron activity (Leslie et al., 2004; Lee et al., 2006; Pfeifer et al., 2008; Likowski et al., 2012). Accordingly, our first regulation condition was conceptualized as requiring an increase in emotional response to faces and involved the voluntary imitation (IMT) of the seen expressions. In contrast, our second regulation condition implied a reduction in emotion response to faces and required expressive suppression (eSUP). We were particularly interested in this emotion regulation strategy (over cognitive re-appraisal) because it provided a more direct comparison with instructed facial mimicry during the IMT condition with regard to the involvement of sensory-motor processes. In addition, a third experimental condition required gender decision (GND) and served as a control task, during which participants were exposed to the same dynamic facial expressions without any explicit demands for eSUP or IMT.

We anticipated stronger responses in brain regions typically associated with emotion processing during IMT relative to the eSUP regulation condition (and possibly GND), because the latter should act to down-regulate the spontaneous neural activity related to emotion processing. We also predicted stronger activation in motor/mirror networks during IMT as compared with eSUP (and possibly GND), due to the fact that this condition should directly affect overt behavioral responses to emotion signals seen in others. By contrast, we anticipated increases in prefrontal cortical activity during eSUP (as compared to IMT) due to stronger demands on cognitive control.

## Materials and Methods

### Participants

The study group consisted of 20 healthy volunteers (12 women, mean age 33.5 ± 4.5 years; mean years of education: 14.9 ± 2.5) with no history of alcohol or drug abuse, major psychiatric disorders (major depression, psychosis, untreated bipolar disorders), head trauma, other neurological disorders, or systemic illness. The local ethics committee approved the study, and all subjects gave written informed consent for their participation in accordance with the Declaration of Helsinki.

### Stimuli

We selected stimuli out of the Geneva Multimodal Emotion Portrayals (GEMEP) database consisting of dynamic multimodal emotion expression video recordings performed by actors (Baenziger et al., 2012). A subset of 30 videos including eight actors (four women) expressing either happiness/amusement (15 videos) or sadness/despair (15 videos) was retained. Only the visual features of videos were presented to participants (no audio was played). The video choice was based on results of an independent validation study evaluating the level of emotional intensity (on a four-point scale) and the accuracy of emotion judgment (rate of correct identification of emotions by independent raters) for each video (Schlegel et al., in preparation), ensuring that our target emotions were easily identifiable by study participants. For selected videos, the mean values of emotion intensity ratings were 3.5 ± 0.2 for happiness/amusement and 2.5 ± 0.4 for sadness/despair (*t* = −8.26, *p* < 0.001, paired *t*-tests); the mean recognition rate was 0.79 ± 0.09 for happiness/amusement and 0.61 ± 0.19 for sadness/despair (*t* = −3.37, *p* < 0.001, paired *t*-tests). The mean duration of movies was 2223 ms (minimum: 1290 ms, maximum: 3970 ms, balanced across conditions).

Videos were then distributed into three different lists, each containing 10 videos counterbalanced for valence (five positive and five negative), and comparable for intensity [*F*(2,29) < 1] and recognition rate [*F*(2,29) < 1]. Each list was used once in each of the experimental conditions described hereafter.

### Experimental conditions

Three experimental conditions were proposed to participants in a block design: (A) *Gender decision* (GND), during which participants had to indicate the actor’s gender by a button press after each video without any particular instruction given to participants regarding actor’s facial expressions; (B) *IMT*, where participants were instructed to mimic actors’ facial emotions during the exposure to emotional stimuli; and (C) *eSUP*, during which participants were requested to voluntary suppress any IMT/facial movement while seeing emotional facial expressions.

Each task was performed three times by participants with conditions presented in a counterbalanced order (ABC, BAC, BCA) during three scanning runs, with the only constraint that IMT always preceded eSUP. Total task duration was 14 min (each run lasted approximately 4 min, 40 s).

### Procedure

Participants were instructed about the different tasks before entering the scanner. During the scanning session, they first saw an instruction slide for 10 s telling them which task was to be performed next. Subsequently, they were exposed to 10 videos per task, each preceded and followed by a fixation cross, jittered between 4159 and 5924 ms (mean 4860 ms). During GND, participants had to indicate the actor’s gender by button press while seeing the fixation cross immediately following emotional videos. During IMT and eSUP, participants either mimicked or suppressed any facial expression during the movie presentation, and then relaxed during the fixation cross periods, with no response required.

### Image acquisition

MRI data were acquired on a 3-T whole-body scanner (Siemens TIM TRIO), using a 32-channels head-coil. For each participant, a structural image was obtained with a MPRAGE T1-weighted sequence [TI/TR/TE/FA = 900/2300/2.98 ms/9°, parallel acquisition (GRAPPA) with acceleration factor 3, FOV = 256 mm × 256 mm, Matrix = 256 × 256, 160 slices, thickness = 1 mm]. Functional images [EPI, gradient echo sequence, TR/TE/FA = 2200/30 ms/90°, parallel acquisition (GRAPPA) with acceleration factor 2, FOV = 216 mm × 216 mm, matrix = 72 × 72] covered the whole brain, were composed of 35 contiguous 3.0 mm axial slices parallel to the inferior edge of the occipital and temporal lobes, and were acquired continuously for a total of 393 images per participant (131 images per session – including instructions, etc.).

### MRI analysis

Image processing was performed with SPM8 (www.fil.ion.ucl.ac.uk) using standard procedures for realignment of the time-series, slice-timing correction, normalization to a standard brain template in MNI space, resampling to 2 mm^3^, and smoothing with an 8-mm FWHM Gaussian kernel. Statistical analysis was performed using the general linear model implemented in SPM8, with a separate regressor for each event type in an event-related manner. For each task (GND, IMT, eSUP), two event types were modeled for each participant (positive and negative faces), using the three scanning runs at the single-subject level. Movement parameters from realignment corrections were entered as additional covariates of no interest for each scanning run, in order to account for residual movement artifacts after realignment. Statistical parametric maps were then generated from linear contrasts between the different conditions in each participant, for each task separately.

Second-stage random-effect analysis was performed using one-sample *t*-tests on contrast images computed in each subject for each comparison of interest. This included IMT > GND, IMT > eSUP, as well as eSUP > GND and eSUP > IMT contrasts, with a statistical threshold of *p* < 0.001 uncorrected and *k* ≥ 20 (Lieberman and Cunningham, 2009). The contrasts GND > IMT and GND > eSUP were not considered because our aim was to investigate the neural substrates of explicit social emotion regulation by comparing emotion expression (IMT) versus eSUP, the condition (GND) during which incidental emotion regulation possibly took place only serving as a control task. Because no significant effects emerged for the eSUP contrasts during these analyses at *p* < 0.001 and *k* ≥ 20, we lowered the threshold to *p* < 0.005 and *k* ≥ 20 for the two eSUP > IMT and eSUP > GND comparisons, in accord with the exploratory nature of this study (for rationale to use similar thresholds in social affective paradigms, see Lieberman and Cunningham, 2009). Finally, raw activation (betas) in functionally defined regions of interests (ROIs) was extracted for all significant voxels and for all three experimental conditions, and the presence of possible activation differences as well as valence effects [positive (POS) versus negative (NEG) – happiness versus sadness] was assessed with paired *t*-tests using a separate statistical software (IBM SPSS Statistics 19).

## Results

The computation of the main contrasts of interest and follow-up statistical analyses revealed the following activation patterns. For a summary, please refer to Table 1.

**Table 1:** **List of activations for the contrasts of interest**.

Number of voxels	*Z*-value	*x*, *y*, *z*	Region	BA
**IMITATION > GENDER**				
660	4.15	6, −2, 58	SMA	6
102	3.66	−52, −2, 50	Precentral gyrus left	4/6
70	4.12	−18, 10, 2	Putamen left	
**IMITATION > EXPRESSIVE SUPPRESSION**				
1980	5.42	−50, −18, 18	Parietal lobe/insula/putamen left	2/3/4/6/40/41/43/44/45/47
838	4.9	54, −26, 24	Parietal lobe/insula left	3/4/6/22/40/41/43/44
316	4.66	8, 16, 36	Dorsal cingulate cortex	24/32
750	4.36	22, −66, 18	Cuneus/precuneus right	18/31
72	3.97	50, 2, 52	Precentral gyrus right	6
20	3.42	40, 32, 4	Anterior insula right	
52	3.42	42, −10, 36	Postcentral gyrus right	6/43
**EXPRESSIVE SUPPRESSION > GENDER (*p* < 0.005)**				
147	3.26	32, −12, 54	Precentral gyrus right	4/6
**EXPRESSIVE SUPPRESSION > IMITATION (*p* < 0.005)**				
22	4.3	38, 24, 52	Dorsolateral prefrontal cortex right	8
35	3.31	30, 66, 12	LPFC right	10

*Coordinates are given in MNI space and associated with Brodmann areas (BA). *Z*-scores represent the numbers from the unit normal distribution (mean = 0, SD = 1). *x*, *y*, *z* coordinates refer to the voxel with highest statistical significance within a cluster. SMA, supplementary motor area; LPFC, lateral prefrontal cortex*.

**Figure 1:**
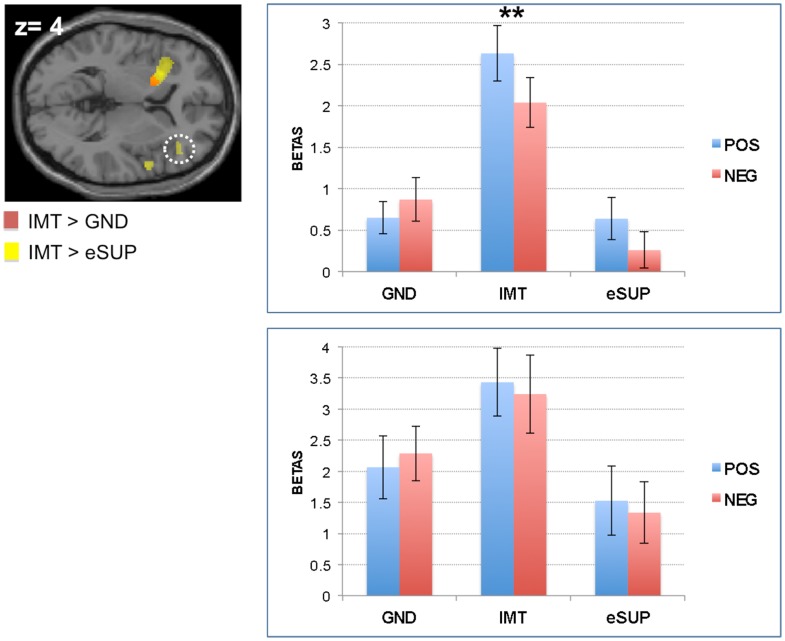
**Modulation of brain areas associated with affective processes**. *Left*: statistical parametric map of bilateral anterior insula and left putamen activity for the contrasts IMT > GND (red) and IMT > eSUP (yellow) at *p* < 0.001 and *k* ≥ 20, superimposed on a template single-subject anatomical brain (T1). *Right Top Panel*: activation values (betas) extracted from the left putamen for all conditions, separated by valence. *Right Lower Panel:* activation values (betas) extracted from the left anterior insula for all conditions. POS, positive/happiness; NEG, negative/sadness. Note that an alternative interpretation of putamen activity is that it was rather directly involved in the motor component of the IMT task (see text). **p < 0.01; activation values (GLM regression weights) are displayed with ±1 SEM.

During IMT (as compared to eSUP and/or GND), neural activity was found increased in several cortical and subcortical brain areas. This included bilateral aINS and left putamen (PUT; Figure 1). In these areas, activity was selectively increased during IMT as compared to both eSUP (as by the fMRI contrast) and GND (*t*s > 2.65, *p*s < 0.016). The putamen (but not the insula) showed a significant valence effect (POS > NEG) during IMT (*t* = 2.87, *p* = 0.01).

**Figure 2:**
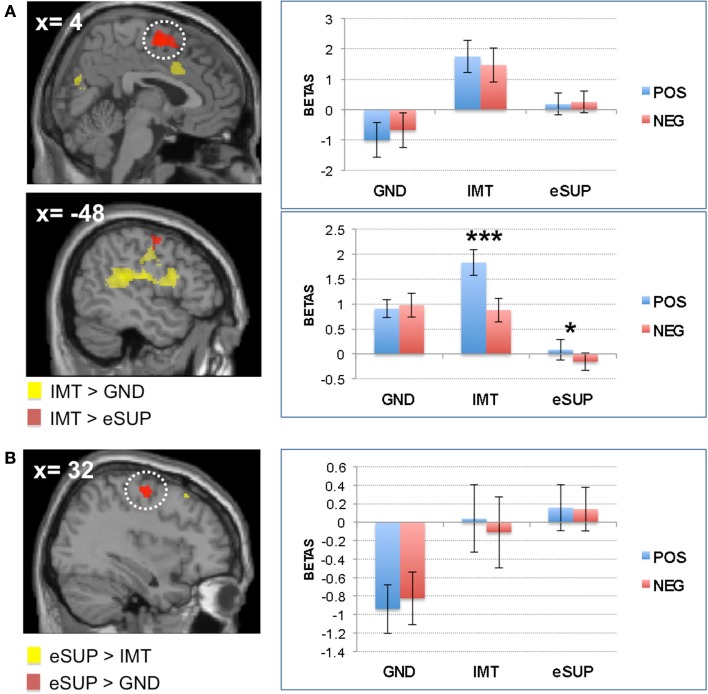
**Modulation of brain areas with (pre)motor, somatosensory, and “mirror neuron” functions**. **(A)** *Left*: statistical parametric map of supplementary motor area (SMA; Top Panel) and left inferior parietal lobe (Lower Panel) activity for the contrasts IMT > GND (yellow) and IMT > eSUP (red) at *p* < 0.001 and *k* ≥ 20, superimposed on a template single-subject anatomical brain (T1). *Right Top Panel*: activation values (betas) extracted from the SMA for all conditions, separated by valence. *Right Lower Panel*: activation values (betas) extracted from the left parietal lobe (yellow cluster) for all conditions, separated by valence. **(B)** *Left*: statistical parametric map of right pre-central gyrus (PCG) activity for the contrasts eSUP > IMT (yellow) and eSUP > GND (red) at *p* < 0.005 and *k* ≥ 20, superimposed on a template single-subject anatomical brain (T1). *Right*: activation values (betas) extracted from the PCG for all conditions, separated by valence. POS, positive/happiness; NEG, negative/sadness. *p < 0.05, ***p < 0.001. Activation values (GLM regression weights) are displayed with ±1 SEM.

**Figure 3:**
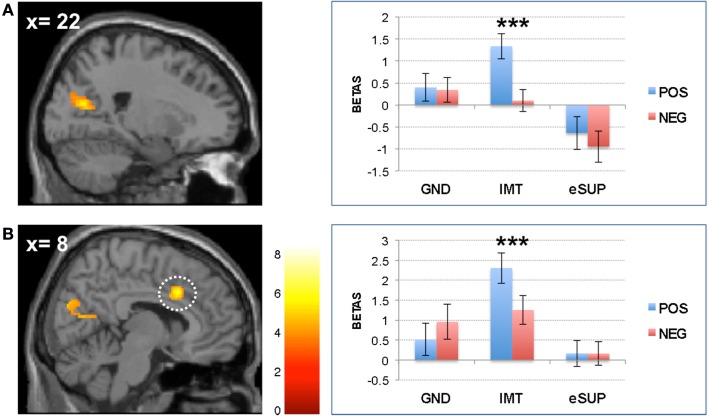
**Modulation of brain areas associated with social cognition/theory of mind and self-monitoring**. **(A)** *Left*: statistical parametric map of (pre)cuneus activity for the contrast IMT > eSUP at *p* < 0.001 and *k* ≥ 20, superimposed on a template single-subject anatomical brain (T1). *Right*: activation values (betas) extracted from the (pre)cuneus for all conditions, separated by valence. **(B)** *Left*: statistical parametric map of dorsal cingulate cortex (DCC) activity for the contrast IMT > eSUP at *p* < 0.001 and *k* ≥ 20, superimposed on a template single-subject anatomical brain (T1). *A*ctivation values (betas) extracted from the DCC for all conditions, separated by valence. POS, positive/happiness; NEG, negative/sadness. ***p < 0.001; activation values (GLM regression weights) are displayed with ±1 SEM.

In addition, fMRI signal was increased during IMT in supplementary motor area (SMA; Figure 2A upper panel) when compared to GND, and in bilateral parietal lobe (extending to pre- and post-central gyri; Figure 2A lower panel) and dorsal cingulate cortex (DCC; Figure 3B) when compared to eSUP. In the SMA, subsequent ROI analyses confirmed that BOLD signal change during IMT was stronger not only relative to GND (as by the fMRI contrast), but also to a weaker degree relative to eSUP (*t* = 2.83, *p* = 0.011), but there was no effect of valence (*p*s > 0.20). In bilateral parietal lobe, the *post hoc* ROI analysis revealed a general consistent valence effect (POS > NEG) during IMT (*t*s > 2.29, *p*s < 0.033), and a similar valence effect during eSUP (*t* = 2.25, *p* = 0.037) in left inferior parietal lobe specifically (Figure 2A lower panel). The DCC also exhibited a selective valence effect (POS > NEG) during IMT (*t* = 4.11, *p* = 0.001; Figure 3B).

Finally, activity was also higher during IMT as compared to eSUP in the (pre)cuneus (CUN; Figure 3A). Follow-up analyses showed that this activation did not significantly differ between IMT and GND (*t* = 1.40, *p* = 0.18), but was significantly increased during both GND and IMT as compared to eSUP (*t* > 3.09, *p* < 0.006 for *post hoc* test). Furthermore, there was a significant valence effect (POS > NEG) in this region during IMT (*t* = 5.99, *p* < 0.001).

**Figure 4:**
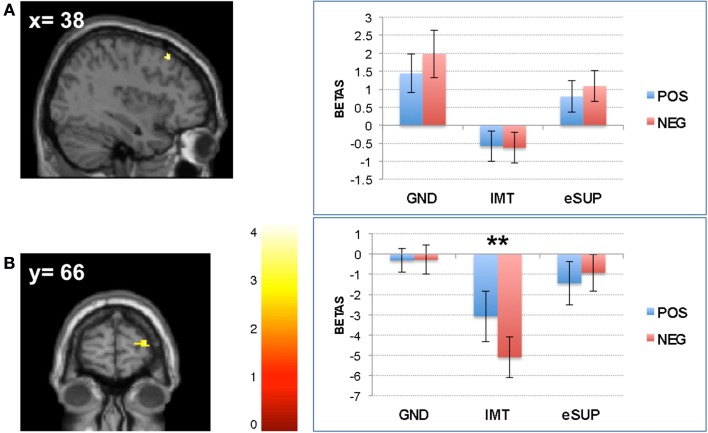
**Modulation of prefrontal cortical areas**. **(A)** *Left*: statistical parametric map of right dorsolateral prefrontal cortex (DLPFC) activity for the contrast eSUP > IMT at *p* < 0.005 and *k* ≥ 20, superimposed on a template single-subject anatomical brain (T1). *Right*: activation values (betas) extracted from the DLPFC for all conditions, separated by **(B)** *Left*: statistical parametric map of right lateral prefrontal cortex (LPFC) activity for the contrast eSUP > IMT at *p* < 0.005 and *k* ≥ 20, superimposed on a template single-subject anatomical brain (T1). *Right*: activation values (betas) extracted from the LPFC for all eSUP conditions. POS, positive/happiness; NEG, negative/sadness. **p < 0.01; activation values (GLM regression weights) are displayed with ±1 SEM.

Conversely, during eSUP, we observed increased activity in right precentral gyrus (PCG; Figure 2B) when compared to GND, as well as in right dorsolateral and lateral prefrontal areas [(D)LPFC; Figures 4A,B] when compared to IMT. In the PCG, activity was actually higher not only during eSUP (as of the fMRI contrast) but also during IMT as compared to GND (*t* = 2.52, *p* = 0.021), but there was no effect of valence. In both the DLPFC and LPFC, activity was again not only higher during eSUP (as of the fMRI contrast) but also higher during GND as compared to IMT (*t*s > 3.03, *p*s < 0.007). In addition, there was a selective valence effect (POS > NEG) in LPFC during IMT (*t* = 3.43, *p* = 0.003).

## Discussion

This fMRI study investigated the neural substrates of social emotion regulation processes by contrasting activity elicited during voluntary IMT versus eSUP to dynamic facial signals of either happiness or sadness. These two regulation strategies were compared to a control condition requiring face gender judgments (GND), which did not involve any explicit voluntary strategy, but maybe incidental regulation based on distraction (see McRae et al., 2010; Kanske et al., 2011; Payer et al., 2012). We found both distinct and shared brain systems for IMT and eSUP effects. These can schematically be regrouped into four different core components of social cognitive affective systems, including (i) affective processes, (ii) somatosensory, (pre)motor, and motor mirror neuron activity, (iii) social cognition/theory of mind, and (iv) executive function. These four domains are not meant to be exclusive, and actually show some overlap, but they represent a convenient framework to summarize and interpret our findings (see Lee et al., 2006; Lieberman, 2007 for similar accounts).

### Affective processes

Two brain areas typically implicated in emotion were modulated by task demands during IMT, namely bilateral aINS and left putamen.

The aINS has been linked with a variety of emotional processes, including emotional conflict and self-reflection (e.g., Lieberman, 2007), as well as feeling states, affective predictions, and empathy (e.g., Lee et al., 2006). Such mechanisms appear recruited when passively observing emotional expressions of others, suggesting the existence of spontaneous mirroring, mimicry, and/or emotion elicitation effects which may operate with considerable automaticity (Leslie et al., 2004; Lee et al., 2006; Dimberg et al., 2011; Likowski et al., 2012). However, in our study, we found selective increases during IMT as compared to eSUP, but did not observe any activation difference during GND relative to eSUP (as could be expected for automatic mimicry during the GND condition). This suggests that spontaneous IMT, which possibly occurred to a certain degree and modulated other brain areas (see next sections), was either insufficient to elicit activation in aINS when attention was directed to non-emotional information in the GND task, or insufficiently inhibited when overt mimicry was suppressed during eSUP. Most remarkably, aINS activity was significantly lower in our study when participants were instructed to apply an eSUP strategy, so as to reduce bodily – and especially facial – reactions to emotions observed in others, by contrast with greater activation during IMT. These results suggest that eSUP may be effective in diminishing some core affective processes mediated by the insula, particularly in social settings that may otherwise involve IMT of expressions (see below).

Interestingly, a similar pattern of selective activation during IMT (relative to both GND and eSUP) was observed in the (left) putamen. The putamen was more activated when participants were told to explicitly mirror the observed emotional facial expressions (IMT) – rather than when just passively observing the face videos during the GND control task – or when instructed to hold their own face still during the eSUP task. Furthermore, putamen activation during IMT was characterized by a positivity bias. Activity in this area has been suggested to mediate approach motivation and represent reward (O’Doherty, 2003; Delgado et al., 2005; Lee et al., 2006), as well as to correlate with stronger zygomaticus reactions to happy faces during spontaneous facial mimicry (Likowski et al., 2012). Therefore, one interpretation is that such activation increases may contribute to the establishment of a successful social connection with another person, which is facilitated when a (positive) emotional expression by the interaction partner can be reciprocated. Alternatively, the selective increase in putamen activity during IMT might signify an important contribution of the basal ganglia to the motor programming and execution of facial movements (Monchi et al., 2006). This would be consistent with the stronger motor demands during IMT relative to the other two conditions, and accord with similar activation patterns in somatosensory and (pre)motor cortex (see next section).

Taken together, these findings corroborate previous results suggesting that eSUP can be effective in down-regulating emotional brain responses under some circumstances (Vrticka et al., 2011), and thus bolster the notion that this regulation strategy should not be regarded as necessarily detrimental or ineffective (Gross, 1998, 2002; Goldin et al., 2008). However, the link between brain activity during eSUP and the behavioral effects of emotion regulation still remains incompletely understood (Goldin et al., 2008; Vrticka et al., 2011). More detailed investigations are also needed to clarify the exact nature of any beneficial role of eSUP, especially concerning its long time consequences (McRae et al., 2010).

### Somatosensory, (pre)motor, and motor mirror neuron activity

Our results also demonstrated significant activity increase during IMT in somatosensory (pre)motor cortex as well as several areas possibly associated with motor “mirror neuron” functions. These sensori-motor effects are consistent with the fact that participants decoded, mirrored, and received somatosensory feedback from the emotional facial expressions they saw and mimicked during the IMT task (Leslie et al., 2004; Lee et al., 2006; Likowski et al., 2012). It is noteworthy that an uniform bias with greater increases for happiness versus sadness was present in both the somatosensory and (pre)motor cortex (BA 3, 4, 6) during IMT, similar to the pattern observed in the left putamen (see above). This positivity bias might reflect the natural tendency to more readily echo positive expressions, such as smiles and laughs, rather than negative expressions (Niedenthal et al., 2010).

BOLD signal change differences between the three experimental conditions were also observed in other premotor regions. On the one hand, the SMA showed significantly stronger activity during IMT as compared to both eSUP and GND. Yet, previous studies reported that SMA may also be involved in motor inhibition (Vrticka et al., 2011; Tabu et al., 2012), and that these effects are enhanced by emotional cues (Sagaspe et al., 2011). Consequently, more research is needed to further determine the role of the SMA in emotion IMT versus regulation, independent of any possible task effects. On the other hand, activity in several sensori-motor regions was significantly different only for the contrast IMT > eSUP, but not IMT > GND, except for an area of the right precentral gyrus (PCG; BA 4/6). Although more direct evidence still needs to be obtained in future studies, this activation pattern is consistent with the possibility that at least some (automatic) motor mimicry/mirroring did occur during GND (especially in left BA 4/6), but that it was not strong enough to elicit emotional activity (see above). Finally, in one region of the right PCG (BA 4/6), activation solely significantly differed for eSUP > GND, but not for eSUP > IMT. This suggests that key parts within the sensori-motor system may also play an important role in behavioral inhibition, besides motor preparation or execution during IMT.

Taken together, our results for somatosensory, (pre)motor, and motor “mirror neuron” seem to be consistent with the regulation needs of each task condition, suggesting that participants properly imitated emotional facial movements during IMT. In turn, motor activity, particularly in the left precentral gyrus, seems indeed to be prevented during eSUP.

### Social cognition/theory of mind

We also observed activity increase in the (pre)cuneus (CUN) during IMT (as compared to eSUP). This region has been associated with a wide variety of integrative tasks, including visuo-spatial imagery, episodic memory retrieval, first-person perspective taking, and experience of agency, as well as theory of mind (Cavanna and Trimble, 2006). Interestingly, the CUN has also been shown to be recruited during the encoding of two-person cooperative behavior (Leube et al., 2012). Similar to affective processes and sensori-motor activation (see above), self and other representations might be particularly activated in the condition when participants were instructed to actively mimic facial expressions displayed by others (IMT). However, such processing was not significantly different during IMT as compared to GND, suggesting that these aspects of social cognition do not necessarily require voluntary expression (Likowski et al., 2012). Also, the CUN was more active for happiness than for sadness during IMT, an effect we previously associated with the possible explanation of happiness being easier to share with others during social encounters (see *Affective Processes* above). The fact that CUN activation was down-regulated during eSUP (as compared to IMT) suggests that behavioral inhibition (instruction not to mirror the facial expressions displayed by others) could also lead to less implication of theory of mind mechanisms, and not only to blunted affective and sensori-motor processing (see above). Such data accords with previous findings in the posterior cingulate cortex/CUN during another fMRI study, where responses to social (versus non-social) visual scenes, possibly reflecting mentalizing processes, were also negatively affected by emotion regulation, although more strongly by cognitive re-appraisal than eSUP (Vrticka et al., 2011). The current observation that CUN activity was reduced by eSUP during viewing of facial expression is consistent with the notion that this emotion regulation strategy might also partly operate by changing the recruitment of cognitive representations associated with mentalizing. Yet, future research is required as to elucidate the differential processes related to the effects of cognitive re-appraisal versus eSUP on these regions, especially concerning the (possible negative) consequences of eSUP on mentalizing.

### Executive functions

Finally, significant differences between conditions were observed in two brain are as typically linked with executive functions.

On the one hand, activity was increased during IMT (as compared to eSUP) in dorsal cingulate cortex (DCC), a region often associated with task monitoring, conflict detection, and adjustment in cognitive control (Carter and van Veen, 2007; Shackman et al., 2011). Hence, the DCC might have been activated when participants were engaged in actively mirroring facial expressions in order to imitate them, as this may have required more elaborate monitoring processes to compare observed and subjectively produced emotional displays. Once more, such mechanisms were enhanced during the IMT of happiness versus sadness. Moreover, there was also a significant difference in DCC during IMT as compared to GND, indicating that such executive control related to emotional social processing was less pronounced during simple observation and attention to face gender.

On the other hand, during eSUP (as compared with IMT), we observed increased activity in right dorsolateral and right lateral prefrontal cortex (DLPFC and LPFC, respectively), encompassing BA 8 and 10. The (D)LPFC has previously been associated with voluntary employment of both cognitive (re-appraisal) and behavioral (eSUP) strategies for emotion regulation, and more generally mediates a variety of attentional and inhibitory processes (Ochsner et al., 2002, 2004; Ochsner and Gross, 2005; Kim and Hamann, 2007; Goldin et al., 2008; McRae et al., 2010; Vrticka et al., 2011). The fact that (D)LPFC activity was significantly stronger during eSUP as compared to IMT thus potentially represents the source of inhibitory activity deployed by the eSUP task. Remarkably, activity in (D)LPFC was also increased during GND as compared to IMT. This pattern is consistent with the notion that some form of automatic or incidental emotion regulation processes may occur through cognitive top-down control during spontaneous viewing or non *a priori* emotional conditions such as our GND task here.

We note that the right LPFC showed a positivity bias during the IMT condition, because activity was decreased to a greater extent for sad as compared to happy movies. As already reported previously (e.g., Vrticka et al., 2011), prefrontal cortical activity is not only elevated during down-regulation, but also recruited during up-regulation of emotions. This pattern might therefore suggest that, in addition to regulatory control during eSUP and GND tasks, this region may also contribute to some regulation mechanisms necessary for sustaining IMT.

In sum, our data suggest that face expression control, in reaction to other faces, might recruit two distinct types of cognitive processes. This includes monitoring and adjustment mechanisms implemented by DCC activation, particularly revealed during IMT, as well as behavioral inhibition subserved by the right (D)LPFC, especially during eSUP. Overall, this is consistent with the notion that prefrontal cortical activity during emotion regulation has many functions, comprising monitoring as well as inhibitory (down-regulation) and facilitatory (up-regulation) processes.

### Limitations

One possible limitation of the present investigation is that the GND condition could involve some incidental emotion regulation in order to focus on face gender (see Hariri et al., 2003). However, such incidental processes may be intrinsic to many other “baseline” or “natural” viewing conditions used in emotion regulation studies. Likewise, our IMT condition might imply some degree of emotion up-regulation through mimicry and facial feedback (e.g., Soussignan, 2002). This overlap between strategies could explain why we did not observe significant effects for eSUP at *p* < 0.001 and therefore had to use a slightly more liberal threshold for some contrasts. Such a lower significance threshold due to the recruitment of the same brain regions by different up- and down-regulation processes is also common in emotion-regulation research (Ochsner et al., 2012).

Another potential limitation concerns the inclusion of both female and male participants in this study, because there are known sex differences in emotion perception and regulation (McRae et al., 2008). Here, due to the small sample size and our main focus on regulation mechanisms, we did not perform any systematic categorical distinction between females and males. In future experiments, however, such sex differences could be addressed more specifically by including a larger number of participants of each group. In any case, our study is the first to systematically compare IMT and eSUP of emotional expression in response to facial displays, and provides novel insights on the neural substrates mediating these effects.

## Conclusion

This fMRI study investigated the neural substrates of social emotion regulation during the exposure to dynamic happy and sad facial expressions by directly comparing active emotion IMT and eSUP. Distinct activation patterns were revealed in brain circuits typically involved in emotion, somatosensory, and (pre)motor processing, social cognition, as well as executive functions. IMT, as compared to eSUP, produced increased activity within all these networks except for those associated with cognitive control functions. In turn, eSUP relied on right precentral gyrus and prefrontal cortical activity, but only the latter region displayed specific BOLD signal increase as compared with possible incidental emotion regulation during the gender (GND) task. Furthermore, we observed a consistent positivity bias (happiness > sadness) in neural responses during voluntary IMT across several brain areas, in line with greater propensity to echo with positive social signals. Altogether, our findings reveal both common and specific activation patterns in networks that mediate emotion expression, IMT and suppression, and therefore add to our knowledge on brain mechanisms that may mediate appropriate social emotional expressions during social interactions.

## Conflict of Interest Statement

The authors declare that the research was conducted in the absence of any commercial or financial relationships that could be construed as a potential conflict of interest.
